# A Qualitative Co‐Production Study of a Solutions‐Focused Approach to Understanding the Barriers and Facilitators to Engagement of Male Suicidal Prisoners in Psychological Therapy for Suicide Prevention

**DOI:** 10.1111/hex.70821

**Published:** 2026-08-16

**Authors:** Yvonne Awenat, Rebecca Crook, David Honeywell, Charlotte Lennox, Dawn Edge, Patrcia Gooding, Gillian Haddock, Helen Brooks, Caroline Hendricks, Daniel Pratt

**Affiliations:** ^1^ Division of Psychology and Mental Health, School of Health Sciences, Faculty of Biological, Medical and Health Sciences, University of Manchester, Manchester Academic Health Science Centre, MAHSC University of Manchester Manchester Lancashire UK; ^2^ Greater Manchester Mental Health NHS Foundation Trust, Suicide Risk and Safety Research Unit, Manchester, Manchester Academic Health Science Centre, MAHSC University of Manchester Manchester Lancashire UK; ^3^ Department of Public Health, Policy & Systems, Institute of Population Health University of Liverpool, Pembroke Place Liverpool Merseyside UK; ^4^ Division of Nursing, Midwifery and Social Work, School of Health Sciences, Manchester Academic Health Centre University of Manchester Manchester Lancashire UK

**Keywords:** co‐production, engagement, prison, psychological therapy, qualitative, suicide

## Abstract

**Introduction:**

Psychological therapies are rarely available for UK suicidal prisoners. A pilot feasibility clinical trial of Cognitive Behavioural Suicide Prevention therapy showed that it was possible to deliver therapy offering the potential to improve outcomes for suicidal prisoners. However, there were challenges to engaging male prisoners in therapy as many experienced difficulties in accessing and talking about their emotions. Difficulty in talking about emotions is common amongst male prisoners, yet this is essential for successful engagement in a talking therapy. The current co‐production study aimed to further understand the contextual influences that impact male prisoners' ability to engage with a psychological intervention focused on suicide prevention and to explore user‐generated solutions.

**Methods:**

Community‐residing male ex‐prisoners with past experience of suicidal thoughts or behaviour during imprisonment participated in individual qualitative interviews followed by a series of six focus groups. Due to COVID‐19 restrictions in place at that time, recruitment and data collection were conducted by remote methods. Data were analysed according to the principles of reflexive thematic analysis.

**Results:**

Fifteen individuals participated: twelve completed individual interviews, ten participated in the focus groups, with seven individuals participating in both.

Three main themes were formed.

1. **Negotiating prison personas and identities** – Pressures to hide emotions, act tough, and distrust professionals.

2. **Command and control in prison** – Prison regimes impact on interpersonal relationships with staff and other prisoners to worsen suicide risk, with prisoner's healthcare needs often neglected.

3. **Making therapy accessible and acceptable** – Prisoners must be able to trust a compassionate therapist for engagement to be successful.

**Conclusion:**

This study epitomises the feasibility and value of meaningful co‐production in research involving populations from marginalised groups such as ex‐prisoners. Our results reveal how the combination of individual prisoner and prison institution socio‐cultural contextual influences impact suicidal prisoners' engagement in psychotherapy. User‐defined suggestions are provided to promote development of potential remedies.

**Patient or Public Contribution:**

Co‐production was achieved by the leading role of a salaried peer‐researcher co‐investigator in liaison with members of our Suicide Risk and Safety Research Group (SSRG) comprised of Experts‐by‐Experience (EbEs). Co‐decision making featured throughout all stages of the research, influencing the protocol design and interview schedule. The personal experience knowledge of the peer‐researcher/co‐investigator enabled targeted recruitment strategies and successful engagement of participants. Shared past experiences of prison life and language positively influenced rapport and engagement of participants during data collection, enabling attainment of rich data. Similarly, the peer researcher/co‐investigator's unique personal experiential knowledge ensured a ‘real‐world’ perspective to analysis and co‐authorship of publications.

## 1 Background

Prison suicide is a worldwide health concern representing the largest cause of non‐natural death of male prisoners [1]. Annual prison statistics for England and Wales report a 9% increase in self‐inflicted deaths (88 deaths = 1.0/1000 to 97 deaths = 1.1/1000 per prisoner) in the year period preceding September 2025 [2]. More detailed data of 2025 show a 93% rise from 14 suicide deaths in the 2nd quartile (April–June) to 27 in the 3rd quartile (July–September) [2]. Similar escalations are reported for self‐harm, as during the 12 months preceding June 2025 there were 55,974 (673/1000) incidents of self‐harm by male prisoners [2].

A systematic review and meta‐analysis of risk factors for prisoner suicide investigated 77 studies involving 35,351 suicides, identifying both individual prisoner and organisational prison context factors [3]. The results indicated that experiencing suicidal ideation, history of self‐harm, aggression and violence were the strongest clinical risk factors for suicide. Also important were poor physical conditions of overcrowded cells and insufficient prison staff resulting in prisoners spending long periods confined to cells unable to access social interaction nor meaningful activity [3].

Usual care for suicidal prisoners involves protocolised monitoring via the Assessment, Care in Custody and Teamwork (ACCT) framework [4]. Prison staff commence an ACCT on identifying a prisoner as at‐risk of suicide enabling further assessment, close observation and access to mental healthcare, led by a case manager.

There is currently no universally accepted evidence‐based suicide prevention treatment for prisoners. Psychological interventions including cognitive behavioural therapy (CBT) can reduce suicidal thoughts and behaviour [5]; however, a systematic review and meta‐synthesis of 15 studies comprising 1303 individuals who received standard face‐to‐face CBT found limited efficacy [6]. Greater efficacy is evident with CBT specific to targeting suicidality in both non‐custodial and within prisons settings [7]. Hence, suicide‐targeted CBT is the recommended psychological treatment for suicidal individuals by the UK National Institute for Clinical Excellence (NICE) [8].

Cognitive behavioural suicide prevention (CBSP) therapy [9] is a theoretically founded intervention specifically designed to target suicidality and has demonstrated feasibility and acceptability in several non‐custodial settings and populations [10, 11]. A pilot RCT and feasibility study of CBSP for suicidal male prisoners provided evidence of adequate recruitment and retention of participants and although not powered to address outcomes did demonstrate promising results in reducing suicidal ideation [12]. However, therapist feedback highlighted specific difficulties for around a third of participants who struggled to engage in therapy due to difficulties in accessing and talking about their emotions [13]. Similar challenges were found in a case series study of modified intensively delivered CBSP for male prisoners [14] where some participants required additional time in the preliminary engagement therapy module of CBSP [14]. The ability to access and verbalise emotions is an important requirement for engagement in psychological therapy, and other research alludes to the communication difficulties experienced by some male prisoners. For example, experience of childhood social and emotional deprivation and abuse is common amongst male prisoners [15] being associated with emotional dysregulation including alexithymia [16], which describes difficulty in identifying and talking about emotions. The current study therefore aimed to understand and develop ways to improve male prisoners' engagement in psychological therapies such as CBSP.

The Prevention of Suicide Behaviour in Prisons: Enhancing Access to Therapy research programme (PROSPECT) [17] aims to further develop and evaluate the clinical and cost‐effectiveness of CBSP within a randomised controlled trial and process evaluation [18] involving 360 participants in four prisons across Northwest England. Within the PROSPECT study, CBSP therapy is delivered to eligible suicidal male prisoners as an individualised, formulation‐driven intervention comprising up to 20 talking therapy sessions over a 6‐month period by a CBSP‐trained psychological therapist. The current study aimed to build on the results of our prior feasibility study [12] to further our understanding of how to improve the engagement of male suicidal prisoners in CBSP therapy within the PROSPECT study.

## 2 Co‐Production

End‐user acceptability is essential for successful implementation of novel interventions [19] with increasing evidence of the value of involving people with ‘lived‐experience’ in suicide prevention research [20]. It was therefore important to work in partnership with the intended beneficiaries to ensure their views and needs were understood and built into the research process. Successful implementation of our earlier feasibility study [12] was strongly influenced by the ‘lived experience’ knowledge provided by the study expert‐by‐experience (EbE) group of people who had experienced suicidality during a period of imprisonment [21]. Their collaboration with the research team enabled successful co‐production of ‘prison‐appropriate’ research procedures [21, 22]. We therefore aimed to emulate and extend our previous approach of forming strong collaborative relationships with the ex‐offender community to develop meaningful research with high user‐acceptability. Co‐production is particularly important for ‘seldom heard’ marginalised populations such as suicidal male prisoners [23]. Our co‐production approach was based on the NIHR [24] recommended model incorporating co‐production based on co‐decision making, co‐delivery, and co‐evaluation. The present study had the benefit of an EbE criminologist employed as a salaried co‐investigator who was fully involved in all aspects of the PROSPECT study and directed and led the study Patient and Public Involvement strategy had the benefit of a ‘lived‐experience’ criminologist co‐investigator employed as a salaried peer‐researcher who was fully involved in all aspects of the PROSPECT study taking a leading role in directing and leading the Patient and Public Involvement (PPI) strategy and leading direct interactions with participants for recruitment and data collection. Throughout the PROSPECT study, and particularly within this qualitative study, we have been privileged to have the involvement of a specialist PPI group named the suicide and self‐harm research group (SSRG). All members have experienced feeling suicidal in several different settings, including primary care, acute mental health wards and prisons. Throughout this study, the peer‐researcher worked closely with SSRG members to seek and implement their views in all areas of the research. Along with the peer‐researcher, the prime role of SSRG members was to ensure that our research was grounded in the ‘real‐world’ issues and situations that impact the lives and views of our participants. Hence, their views directed our approach to ensuring ethical participant care, the development of research interview questions and co‐interpretation of the data analysis.

### 2.1 Aims

This study aimed to co‐produce knowledge to understand and overcome the barriers faced by male prisoners in engaging in psychological therapy for suicide‐prevention.

Broad research questions included:

1. What are the contextual issues in prisons that contribute to suicidal male prisoners' difficulties in accessing and engaging in psychological therapy?
2. How can these barriers be removed or minimised?

### 2.2 Ethical Considerations

Ethical approval was obtained from the Health Research Authority Health and Care Research Wales (Reference [20]/SS/0021). All data protection and research governance regulations were upheld. Informed consent was obtained from all participants who were given the option (but declined) identification by a pseudonym.

During the focus groups, participants were requested to respect confidentiality of information shared by others but were advised that researchers could not guarantee adherence by participants. An information sheet signposting participants to sources of further support was provided to all participants along with an invitation to receive a follow‐up debriefing phone call.

## 3 Method

A qualitative method was selected as a natural conversational and interactive way of accessing participants' ‘real‐world’ lived experience knowledge. The study comprised a two‐phase design commencing with individual interviews followed by a series of six synchronous focus groups. All data collection was conducted remotely.

### 3.1 Participants and Settings

We had originally planned to interview male prisoners within the prison setting; however, this was not possible due to the onset of the COVID‐19 pandemic when in‐person contact with external researchers was prohibited. We therefore redesigned our approach to recruit a convenience sample of ‘by‐proxy’ prisoners. Hence, participant eligibility sought adult male community residents with experience of suicidal thoughts or behaviour during past imprisonment in a UK prison, along with access to a smartphone. For maximum inclusivity, no further restrictions were specified. Information about the study was sent to third‐sector organisations known to support prison leavers requesting its circulation via their websites, mailing lists and newsletters.

### 3.2 Recruitment

#### 3.2.1 Phase 1

This generated phone enquiries from 14 individuals who were provided with further information and screened for eligibility, resulting in 12 people providing informed consent to participate in an individual telephone interview.

#### 3.2.2 Phase 2

The same recruitment process was repeated for the Phase 2 focus groups with an additional eligibility criterion requiring access to equipment for online video meetings. Twelve individuals expressed interest, resulting in ten consenting to participant, seven of whom had also participated in the Phase One individual interviews.

### 3.3 Data Collection

#### 3.3.1 Phase 1

A semi‐structured interview schedule was constructed informed by the results of our prior research [12] and further enhanced by the ‘lived‐experience’ knowledge of our peer‐researcher who had also consulted with EBEs from the SSRG. Individual qualitative interviews of up to 1 h were conducted by telephone with an encrypted audio recorder prior to transcription and anonymisation by a university‐approved contractor, after which the research team made quality checks for accuracy.

#### 3.3.2 Phase 2

A series of remote 2‐h synchronous online focus groups were held [25]. A semi‐structured topic guide informed by the findings of the phase 1 interview data aimed to elicit further in‐depth inquiry and to provide some initial structure to stimulate group discussions. Participants were asked to draw on their own personal experiences in considering the situation of a vignette describing a fictitious ‘typical’ persona formed from the combined experiences of the peer‐researcher and co‐authors who implemented the prior feasibility and pilot RCT [12] (see Table 1. Focus Group Vignette). The number of focus groups was not pre‐determined but employed a pragmatic approach based on research team judgements of data sufficiency [26] in addressing the research aims. This resulted in six focus groups being held at monthly intervals.

**Table 1:** Focus group vignette.

*‘Mark is a 30‐year‐old male who has recently been sentenced to 5 years in prison for GBH. He has a history of self‐harm and since being in prison has expressed suicidal thoughts. Before coming into prison, Mark had never accessed any support for his mental health concerns because he doesn't like talking about his emotions as he finds it hard to communicate. Mark also struggles with reading and writing. Mark was brought up in a household were no one talked about feelings or emotions, so he struggles with finding the words to express how he is feeling. The idea of being emotionally vulnerable is worrying to him.’*

The first focus group commenced with introductions, agreeing basic ground‐rules and explaining the researcher's roles. For each focus group, a team member acted as moderator by introducing core questions around the vignette and inviting participant‐generated topics for discussion. Group dynamics were monitored by another team member to avoid researcher dominance, promote equitable participant contribution and to support the moderator in encouraging participant–to‐participant interaction. All researchers made observational field notes, and for each session a specific researcher was allocated to be availability in case a participant encountered technical or personal difficulties. These roles were rotated among the research team for each focus group. Following each focus group, the research team met to review the data and identify pertinent issues requiring further enquiry at the next focus group.

### 3.4 Data Analysis

Analytic interpretations were influenced by relativist ontological assumptions and contextualist epistemology, which accorded with our active role as researchers and EbEs working together in co‐constructing knowledge from data representing multiple realities [27].

The principles of reflexive thematic analysis [28] were applied to the entire qualitative data corpus. An inductive approach to semantic and latent coding enabled identification of codes which were grouped together to form tentative themes. Active involvement of the SSRU group members was led by the staff peer‐researcher who attended monthly SSRU meetings where emerging analytic ideas were shared and discussed. Hence, several analytical iterations occurred prior to confirmation that the final themes represented participants' views of shared meanings.

The process of analysis comprised two separate stages commencing with the data from the Phase 1 individual interviews from which themes representing shared meaning were identified but required further in‐depth inquiry to expand and clarify. To achieve this, a focus group approach was chosen based on the potential for greater inductivity and spontaneity by participant‐to‐participant discussion [25]. Data from each focus group was analysed as soon as possible after collection to identify target areas for discussion in the next focus group. Finally, the findings from both phases were reviewed again to confirm that all pertinent issues had been addressed.

### 3.5 Reflexivity

In accordance with the principles of *reflexive* thematic analysis, we embraced our role as active co‐producers of knowledge rejecting more traditional conceptions where researchers' influence is viewed as a negative bias requiring control [29]. The core team comprised four researchers, two male and two female, with all identifying as White British. Two are clinical academics with extensive experience of suicide prevention research and clinical practice, another is an academic criminologist with lived experience of imprisonment, and another researcher had experience of qualitative research but relatively less experience of working in prison. The range of backgrounds and life experiences of SSRG members, whose collective experiences of suicidality across several areas including primary care, acute mental health wards and prisons further contributed to the breadth and diversity of the lens through which the data was perceived and interpreted.

## 4 Results

### 4.1 Participants

Participants were males aged between 18 and 65 years old, individual ages are presented within age brackets to preserve anonymity (see Table 2). Fifteen individuals participated, comprised of twelve in the Phase 1 individual interviews and ten in the Phase 2 focus groups. Seven participated in both phases. Eight identified as White British, and five as mixed heritage. Two participants declined to provide any demographic information, and one declined to provide age and gender (see Table 2).

**Table 2:** Participant demographic information.

ID: Individual interview	ID: Focus group	Gender	Age bracket	Ethnicity
II:01	n/a	Male	46–55	Mixed race
II:02	FG:02	Male	18–25	White British
II:03	FG:07	D	D	D
II:04	FG:01	Male	46–55	White British
II:05	n/a	D	D	D
II:06	FG:11	Male	26–35	White British
II:07	FG:05	Male	36–45	Mixed race
II:08	n/a	Male	26‐35	White British
II:09	FG:03	Male	56–65	Mixed race
II:10	FG:04	Male	26–55	White British
II:11	n/a	Male	55–64	White British
II:12	n/a	Male	55–64	Mixed race
n/a	FG:08	Male	26–35	Mixed race
n/a	FG:09	Male	55–64	White British
n/a	FG:10	D	D	White British

Abbreviations: D = declined to report, FG =Focus Group, II=Individual interview, n/a = not attended.

## 5 Reflexive Thematic Analysis Findings

Analyses of interview and focus group data revealed both barriers and facilitators to prisoners' engagement in suicide prevention psychotherapy illuminating several important factors to consider. The issue of ‘trust’ underpinned much of the data from which three main themes were formed. Theme 1 comprised data around the personas and identities assumed by prisoners to conceal distressing emotions within the prison environment and how these could impact therapy engagement. Theme 2 encapsulated data specific to the prison establishment and prison regimes, while Theme 3 reflected participants' suggestions for improving engagement in therapy. Illustrative quotes are provided from across the data corpus. Quotes arising from the Individual Interviews are labelled by ‘II’ and those from the focus groups as ‘FG’ followed by the participants' research identity number.

### 5.1 Theme 1: Negotiating Prison Personas and Identities

In this theme participants described the contextual factors of prison life that could influence a suicidal prisoner's behaviour and demeanour consequently impacting on their willingness to access and engage in suicide prevention psychological therapy. For example, irrespective of a prisoner's pre‐imprisonment disposition, concealment of emotionality by portrayal of a tough prisoner ‘persona’ was required to ensure personal safety and acceptance by other prisoners. As such, the tactics necessary for survival in prison could conflict with the ability to seek and engage in psychotherapy for fear of being viewed as weak thereby risking exploitation and harm by other prisoners.

i. Being the ‘macho man’
  Male prisons were described as hyper‐masculine *“alpha environments” (FG1:08)* requiring projection of a tough ‘macho’ demeanour to be seen as strong for gaining the respect and acceptance of fellow prisoners. This was deemed necessary to assure personal safety. Portrayal of strength was achieved by not showing any signs of emotion, because being emotional was perceived as a weakness. Therefore, the unspoken rule in prison was *“never show weakness” (II:10)*.
  I mean in prison, everyone wants to walk around like they're macho men, you know [laughs]. They don't really want to admit that they got, be seen as weak. They all act tough, you know, and not the truth,'cos we've all got feelings and emotions or whatever, but we don't talk about that you know, how they feel or are sad or ‘I need some support’ and all that stuff,'cos that's a weakness.
  (II:12)
ii. Wearing the mask
  A way of hiding negative emotions and preventing exposure of vulnerabilities was described by participants as akin to putting on a mask.
  … In prison a lot of people mask their issues and try and become somebody else or something that they aspire to being in prison, if that makes sense. Like a lot of people put a front on, don't they.
  (II:08)
  Participants warned that such factors, including being concerned about being labelled as weak, could prevent engagement in psychotherapy due to fears of having to remove the ‘mask’ and ‘open‐up’ about their emotions.
iii. The therapist's identity

Participants explained that anyone and everyone other than fellow prisoners is generally classified as outsiders (i.e., ‘them’) being automatically perceived as someone who is aligned to the prison establishment. Hence, concerns were expressed about the therapist's identity regarding their employment affiliation.

Because you imagine when you're a prisoner there's us and then there's them. And that is it, you're just them, you're the screws, you're the probation, you're psychology, you're in that box of ‘them’.

(II:10)

This ‘them and us’ distinction was discussed in the context of participants' prior experiences of being let down by authority figures who therefore could not be trusted with personal information. Hence, in terms of experiencing therapy within a clinical trial where the researcher trial therapists are employed by an external funding institution, the separateness of identity and employment affiliation was seen as advantageous for the development of a trusting therapeutic relationship.

I guess you mentioned earlier that one of the parts is that you're staffing it yourself (refers to RCT therapy staff), you're not relying on people that are already in the establishment. I think initially when you said that I felt like that was the right thing to do. That would have felt good as a sort of service user of that if that was the case… For me, like, the sort of independence of it is something that would appeal to me as a prisoner. It would feel like, if I can go and talk to someone and it's not going to end up on a record, or there isn't going to be an officer outside listening to what's happening and stuff, those things are important to me.

(FG1:02)

#### 5.1.1 Theme 2: Command and Control in Prison

There were two aspects to this theme. Firstly, issues related to the prison environment, its organisational culture, daily structures and routines and secondly, issues specific to the role of the prison officers.

##### 5.1.1.1 Primacy of Prison Regimes

The prison's institutional priority of maintaining order and security was perceived to supersede the healthcare needs of individual prisoners. Such operational priorities were seen as potential barriers to prisoners' participation and engagement in a programme of therapy. Medical and psychological therapy appointments would typically require the prison officer to escort the prisoner from his cell to a treatment room in another department. One participant reflected on the demotivating impact of the uncertainty of whether arranged therapy appointments would actually occur.

And doing that [therapy] and then sort of still be like, you know, enslaved to the sort of operational requirements of the prison at the time as well. And, yeah, building up an expectation of even just having a session and that being broken, you know, it's going to be more difficult to convince them the next time and so on.

(II:02)

Frustration and disappointment of frequently thwarted appointments were cited as a potential threat, likely to weaken a prisoners' commitment to attending therapy

I remember getting some treatments or – I was seeing a psychiatrist once a fortnight when I was at [HMP PRISON], and, you know, it was these half‐hour sessions. They got cancelled quite a lot…

(II:02)

Whilst some participants alluded to the existence of benevolent prison officers who were caring and supportive, others were more critical of the brutality of treatment by prison staff, even doubted the authenticity of prison commitment to suicide prevention.

Like I remember when I was in prison and I would look at young lads that were going through the suicide thing, and I would feel sorry for them ‘cos I'd just think, look, that's what these people want you to do. That's exactly what they want you to do, and that is what is happening in these prisons, you know. People are being pushed into that frame of mind by regimes and by staff.

(FG5:08)

From the ‘tone’ of participants' narratives it was clear that organisational failures to meet individual prisoners' basic health needs were perceived by some to represent the establishments' view of prisoners' insignificance relative to prison organisational priorities. Implicitly this suggested that forces beyond staff workload pressures, more akin to wielding authority, control, and punishment were perceived to be responsible.

##### 5.1.1.2 Role of Prison Officers

As the prison establishment's ‘front of house’ for prisoners, prison officers held great power and control over prisoners' everyday lives. This amounted to literally acting as ‘gatekeepers’ as only prison officers held the power to unlock the cell door and transport prisoners to access the healthcare appointment.

Yeah. I mean, so, I think that's quite a sort of situation where it may be a prison specific thing, but like as with – I'm sure you know, as with anything, you get treated like you are at the whim of the landing officers opening you up, getting you back and stuff, and there are so many things that could stop that happening.

(II:02)

The topic of being denied the basic human right to healthcare generated much heated discussion and validation of similar views and shared experiences among participants. This was perceived as another way that officers imposed their power and control to reinforce the dependence of prisoners on prison staff by participants who understood themselves to be“*… at the mercy of the prison officers” (II:11)*, based on perceptions of the particular prison officer's mood or whim….

like you'll get a healthcare appointment and let's say that's to see the psychologist or a mental health nurse. And if officers aren't feeling particularly energetic that day, they won't take you.

(II:10)

#### 5.1.2 Theme 3: Making Therapy Accessible and Acceptable

This theme represents participant‐generated suggestions to address some of the challenges described in themes 1 and 2 in outlining possibilities for a tailored intervention that sees suicidal prisoners as individuals and can be adapted to their needs.

##### 5.1.2.1 Building Trust by Transparency and Compassion

The cruciality of a trusting relationship with the therapist for successful engagement in therapy was stressed. Trust was perceived as difficult to gain yet the most important and necessary component for engagement. Participants highlighted the essential attributes of therapists to establish and maintain a strong therapeutic alliance valuing therapists who could be *“empathic” (II:11)* yet *“straight talking” (II:06)*.

It's building trust with somebody, building that human connection with somebody and being patient, and over time somebody eventually will relax, make themselves – allow themselves to be vulnerable with you and then that's when it'll [*referring to talking about suicidality*] come out.

(II:06)

Meaningful ways of developing trust were suggested. Participants valued the opportunity to discuss realistic expectations along with the demands and potential gains of therapy with the therapist. Open and honest talk, but also portrayal of basic human compassion was viewed as essential to inspire hope and demonstrate trustworthiness.

You know, a relationship is about building trust. Once you've got a relationship with trust, it should come ‘hand‐in‐hand’, you know, mutual respect, trust. That's how you get a person to talk. That's how you get people to share their emotions, you know, make it easier for them. So, that's just like, you know, informing someone, like you said, with clarity, and being open and honest, you know, about what to expect, not giving them pipedreams or anything. Just tell them how it is, you know. It could be like a torch in a tunnel, just an inkling of hope could be all somebody needs, you know.

(FG4:03)

However, concerns were expressed that therapy might make matters worse, leaving the prisoner alone and vulnerable in a highly emotionally roused state following therapy. The need for absolute privacy and confidentiality of therapy was stressed with other potential sources of support such as talking to prison officers and fellow prisoners distrusted due to the lack of confidence in these being confidential.

Is it likely, going into the session, that I know I'm going to come out feeling bad, and, you know, what's going to happen the other side of it? Are the officers going to know that like I've been in a session that's heavy? Are the other prisoners going to know?

(FG4:02)

Participants offered suggestions of ways of reducing the impact of lack of trust, including the importance of first impressions during the initial meeting with the therapist, this being highlighted as a unique opportunity to demonstrate trustworthiness.

The first session has got to be the practitioner gaining the trust of the inmate. Because what you've got to remember is the inmate, or most inmates, will not trust authority figures or can have problems with trusting authority figures. And sometimes they can see therapy,…, therapy providers or medical staff or any of that, even though they're in a different uniform and they come under a different name, they still see them as someone who can go and sort of grass them to the staff on the wing.

(II:03)

Another suggestion was for the therapist to avoid portrayal of the authoritative manner associated with prison professionals for a more person‐centred informal style conducive to establishing rapport in therapy.

Just getting to know them is better than just expecting them to open‐up to you…. Like it's easier to talk to someone when you see them as a friend. If you see them as a professional, then you're automatically backing up. Everyone in jail,… they're sick and tired of professional people.

(II:05)

##### 5.1.2.2 Need for Understanding Prison Life

Participants recognised that therapists were unlikely to share the experiences of suicidal male prisoners but expressed the importance of having a *“baseline level of understanding” (II:06)* so that those therapy recipients wouldn't have to *“explain the basics before you even really get into the problem” (II:06)*.

Also stressed were the benefits of talking to someone with *“lived experience,”* and how *“powerful” (FG2:06)* it was to speak to someone who, based on their shared experience, really understood suicidal prisoners' situation and needs.

##### 5.1.2.3 De‐Stigmatising Language

The need to scrutinise the words used to describe therapy in the prison environment was raised. Concern was expressed regarding the use of words such as “therapy” and “therapist” due to their association with seeking mental health support, this being a stigmatised behaviour in prisons. The stigma attached to seeking such support would be disapproved of by other prisoners who may label the help‐seeker as an *“idiot”* for *“going to get therapy” (II:01)*.

To overcome these potential language barriers, suggestions were made of alternative words for the therapy *“… I would class it as work or education course or something like that.” (II:03)*.

This topic produced some lively discussion with several suggestions of alternative ways of labelling the therapy and therapist.

Call them a tutor or call them a course leader or something like that. Keep the terminology of a course. So, they're either… they're teaching people life skills aren't they, so they're a tutor for, essentially life skills… So, I think the terminology you use is important, because people will be going in there with preconceived ideas… But they're going into a prison, and from the offset, if you set this up as not being therapy and you set it up as a course, then people will look at it as a course.

(FG3:11)

#### 5.1.3 Illustration of the Results

**Figure 1:**
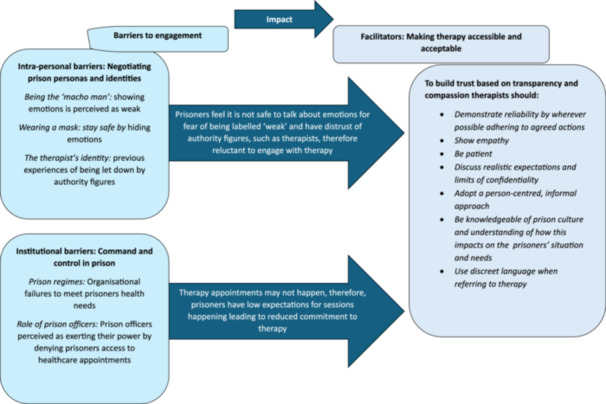
Conceptual model of user‐defined barriers and facilitators in CBSP therapy.

A conceptual model illustrating the above results is presented in Figure 1. This shows how prisoners' intrapersonal barriers combine with the prison institutional barriers to challenge suicidal prisoners' ability to disclose their suicidality to seek and engage in CBSP therapy. This sits alongside user‐defined suggestions of how therapists can facilitate greater accessibility and acceptability to aid suicidal prisoners' engagement in therapy.

## 6 Discussion

### 6.1 Main Findings

This is the first study to report the views and perspectives of male ex‐prisoners not only of the challenges to engaging suicidal prisoners in suicide prevention psychotherapy but also crucially in presenting user‐defined suggested solutions. Importantly, this captures the unique experiences of a marginalised population about the taboo subject of suicide, thereby giving voice to views that are seldom heard. New knowledge obtained directly from ex‐suicidal prisoners outlines the complex interplay of internal and external factors that impact on suicidal prisoners' ability to access and engage in therapy.

The issue of ‘trust’ was seen as a core essential pre‐requisite for engagement in prison‐based suicide prevention psychotherapy, thus impacting at some level across all themes. This accords with prior literature extolling the primacy of a strong therapeutic alliance built on trust [30, 31] being particularly so for psychotherapy with people experiencing suicidal thoughts [30, 31]. Trust is particularly contentious within prisons which have been termed ‘low‐trust’ environments where prison staff are trained to distrust prisoners [32]. Reciprocally, participant narratives described how frequent past breaches of trust by authority figures create a default position of distrust of all professionals, including psychotherapists. Participants understood that the trial therapists within the PROSPECT study [17] were not employed by the prison and this was seen as advantageous. However, beyond the context of the PROSPECT study [17], psychological therapy is already established and delivered by therapists working within prison mental health teams. Although known to encounter huge contextual challenges, research of psychological therapy delivery within prisons also evidences the opportunity for meaningful therapy [32, 33, 34].

Theme 1 revealed the risks that prisoners face in talking about their suicidal thoughts and emotions which would be required to access and engage in therapy. Explanations demonstrated how survival in prison required the prisoner to mask negative emotions and adopt a tough, macho persona. Two issues contribute to understanding this situation. Firstly, the contextual factors inherent within the harsh prison environment itself can underpin and/or interact with prisoners' imported vulnerabilities towards suicidal ideation and behaviour [32]. A systematic review involving interviews with several hundred male prisoner survivors of a near‐lethal suicide attempt demonstrated how the conditions of imprisonment fuelled and precipitated their suicide attempt [35]. Survivors reported views suggesting that had they been able to talk to someone about how they were feeling, the attempt could have been avoided [35].

Secondly, prison socio‐cultural norms mitigate against a suicidal' prisoner expressing emotional distress for fear of this being perceived as weakness by other prisoners who could respond with violence and aggression [32, 34, 36]. Such behaviour can be understood as hegemonic masculinity [36], commonly associated with male prisoners, and epitomised by amplification of male gender roles manifesting as dominance, stoicism, physical toughness and suppression of emotions [37]. Paradoxically such efforts to avoid harm from other prisoners co‐exists with internal thoughts of self‐destruction.

Beyond the intra‐prisoner barriers to engagement in therapy, participants described the institutionally situated physical barriers of thwarted access due to prison officers' failure to escort them from their cell to the appointment venue. Participants attributed such neglect of their healthcare needs as a component of deliberate de‐humanisation of prisoners by prison officers knowingly withholding support. Harvey describes such behaviours as prison ‘deprivations’ by imposing the ‘pains of imprisonment’ within the prisons' punishment function [34]. Uncertainty and doubt about the reality of being able to attend therapy can create low expectations [38] and negatively impact trust and motivation to commence and continue therapy [39]. In such situations, neither prisoner nor therapist has any control. Equally, issues such as workload pressures beyond the control of individual prison officers may underpin their actions as reports of low and unsafe prison staffing levels exist [40]. Nevertheless, for some participants such barriers to prisoners' access to treatment enhanced their distrust of the genuineness of prison officers' commitment to suicide prevention.

Theme 3 outlines participants' ‘lived‐experience’ based suggestions for how therapists could foster a trusting therapeutic relationship with prisoners. The impact of first impressions during initial meetings with the therapist was stressed as concurring with existing evidence of factors predictive of forming a strong therapeutic alliance [38]. Participants stressed the need for the therapist to portray honesty, warmth and respect to connect with the prisoner at a friendly human level to counter prior negative experiences of authoritarian polarised power dynamics. This accords with the approach advocated by experienced prison psychological practitioners who emphasised the value of active listening, showing authenticity and a non‐judgemental attitude [41]. Such qualities mirror the long‐established Rogerian [42] core conditions for therapeutic relationships of genuineness (congruence), unconditional positive regard (acceptance) and empathy (demonstrating appreciation of how the prisoner perceives the situation) [42]. Participants also stressed that therapists must understand the context of prison life and avoid portrayal of professional formality which also concur with Rogerian ‘necessary and sufficient conditions’ of congruence (genuineness), unconditional positive regard and empathic understanding [42].

Literature of the complexity of providing psychotherapy in prisons reaffirms support for Rogerian person‐centred approaches [34] whilst also warning of important adaptations necessary to maintain prisoners' safety during therapy. For example, our participants suggested how the therapist could engage the prisoner in therapy to remove the ‘mask and: “… *allow themselves to be vulnerable with you” (II:06)*. However, therapist participants of Harvey's [34] study warned of the need to assist the prisoner put the ‘mask’ back on before leaving the therapy room”*… you've got with participants to help them put the shields up and refocus them on being somebody else in the prison and get their… get them back into role before they leave the session (* ^*35*^ *p310).*

Participants also raised concerns about safety in therapy and confidentiality which are paramount for a trusting therapeutic alliance. Additional challenges to assuring confidentiality exist in prisons where all professionals are obliged to report disclosures of criminal activity [34], meaning that only ‘limited confidentiality’ can be offered by therapists.

Concerns were also expressed that terms such as ‘therapy’ and ‘therapist’ could deter prisoners from accessing treatment due to the stigma associated with mental health problems and consequent risk of exposing vulnerability to other prisoners. Whilst this is certainly a potential barrier to be considered there are ethical considerations in labelling the intervention and the practitioner as something that they are not.

### 6.2 Strengths

#### 6.2.1 Peer Researcher‐Led Co‐Production

A major strength of this study was the prominent role of a salaried co‐investigator peer‐researcher [36] whose EbE knowledge enhanced the design, delivery and analysis throughout all stages of the research. This was particularly impactful in participant‐facing activities as although ex‐prisoner populations have been termed ‘hard‐to‐reach’ or ‘seldom heard’, we achieved excellent levels of recruitment and retention of ex‐prisoner participants affording strong credibility to the findings. Our experiences of successfully accessing and engaging ex‐prisoners in suicide‐focused research adds to the growing body of literature that challenges historic perceptions of difficulties epitomised by the term ‘hard to reach’ populations', however, more research in this area is required.

#### 6.2.2 Methodological Innovations

The Covid‐19 pandemic imposed unprecedented barriers prohibiting our access to prison‐based participants, thereby necessitating major changes to our research plans, more detail is presented by Pratt and Crook [43]. We had planned to interview current prisoners face‐to‐face but had to change to remote data collection. When preparing for this research we were unable to find precedent literature of exemplars of synchronous sequentially conducted remote online focus groups. Hence, to our knowledge, at the time of its inception this was the first study to include online serial synchronous focus groups with ex‐offenders. So, although remote data collection was not our preferred choice, by embracing this approach we were able to develop successful practices that can be repeated and shared with others.

Other noteworthy advantages included the increased contact with participants by meeting with them in six serial focus groups which afforded the opportunity to develop stronger relationships [44] optimising trust and enabling rich discussions of sensitive issues around suicide. Similarly, the relative anonymity of participating in an online focus group may also have contributed to greater confidence to talk freely about contentious issues experienced as a suicidal prisoner. Arguably, this may have been more restricted within an in‐person group from a smaller geographical area within which participants could identify each other. Lastly, the reduced costs of online research enabled us to draw participants from a much wider geographical area, resulting in a more diverse population and easier participant access from individuals throughout England and Wales.

### 6.3 Limitations

Although the characteristics of study participants were the closest possible to our ideal sample of currently imprisoned males with experience of suicidality, it is possible that recall of their past experiences may be limited, and participants' views and perspectives may have differed from current suicidal prisoners limiting transferability. Background literature refers to the prevalence and relevance of alexithymia within populations of suicidal male prisoners. However, this topic did not feature strongly in participant narratives. Eligibility for participation in this study required experience of suicidal thoughts or acts during a period of past imprisonment. Screening was undertaken to confirm this eligibility requirement, but disclosures of current suicidality would have negated eligibility. Hence, the temporal and environmental distance of our community‐dwelling ex‐prisoner participants from their past positions as incarcerated suicidal prisoners may explain the limited focus and depth of data concerning alexithymic type difficulties. It is also possible that participation in this study requiring the ability and willingness to talk about past experiences of suicidality while imprisoned may not have attracted those who would find this difficult.

### 6.4 Implications for Research and Clinical Practice

This study was primarily conducted to address the challenges of accessing and talking about emotions which negatively impacted the ability of a significant number of suicidal male prisoners to engage in therapy in our prior feasibility study of CBSP therapy. Our results have generated valuable information of great importance to informing the conduct of the present large‐scale multi‐site definitive RCT of CBSP for male prisoners (the PROSPECT study). Specifically, this has enabled the transfer of ‘live’ real‐world research findings to inform our bespoke preparation and training programmes for our research staff and research trial therapists, many of whom have not previously worked in prisons. Translation of these results has enhanced practice in the PROSPECT study by ensuring staff training fosters understanding of how prison and prisoner cultural attitudes and relational dynamics impact on prisoners' ability to engage in recruitment to the study and engage in therapy if so allocated. Similarly, understanding the heightened importance of sensitive communication, privacy and respect for confidentiality is imperative to gain prisoners trust and paramount for their safety in the hostile prison environment. Recognition of the need for scrupulous attention to upholding agreed actions and appointments is important along with understanding that prisoners may fail to attend therapy appointments due to forces beyond their control. This has led to the creation of a new monitoring identifier of Could Not Attend (CAN) alongside Did Not Attend (DNA). To help prisoners maintain their own safety therapists must build in ways of supporting prisoners to ‘put the mask back on’ towards the end of sessions. Our results have also highlighted the need to develop a special therapy engagement resource now in progress.

Beyond our own research, this study also has utility for psychological practitioners working with suicidal prisoners, the wider mental healthcare team and all front‐line prison staff. It is also for prison managerial and commissioning staff, Ministry of Justice, politicians and society itself to consider if current prison regimes are humane and effective in the light of escalating suicide deaths [1, 2].

The remoteness of online research can impede researchers' ability to observe nuanced signs of participant distress [45] hence specific participant care and debrief protocols were developed for this study. As alluded to by Speers et al [46], the lack of a physical geographical venue in favour of remote digital platforms may have facilitated greater confidence for participants to express potentially contentious views formed from past experiences as suicidal prisoners. Similarly, the increased informality of participation from within their own selected venue may have contributed to more flattened hierarchy of power dynamics between researchers and participants [40].

The brutality of the prison culture impacts how prisoners behave to survive their imprisonment by adoption of tough ‘alpha male’ personas [27]. Paradoxically such strategies, despite aiming to ensure personal survival, may ultimately be counter‐productive to survival by inhibiting the ability to seek and access suicide prevention interventions. It is therefore important that all health care staff, particularly those new or unfamiliar with prison culture, look beyond the projected persona to recognise the potentially suicidal person within. The impact of language used to describe therapy and the therapist requires further research.

## 7 Conclusion

Successful engagement of suicidal prisoners in suicide prevention interventions could literally be life‐saving; hence our findings are particularly important for prison mental health care and general healthcare staff who work with suicidal prisoners.

Prisons exist as ‘total institutions’ with rigid procedures and internal cultural norms that present challenges to conducting research and to delivering psychological therapy. The adverse de‐humanising context of prison institutions both fosters suicidality and imposes barriers to accessing and engaging in treatment interventions.

## Author Contributions

**Yvonne Awenat:** conceptualisation, methodology, data curation, writing – original draft, writing – review and editing, investigation. **Rebecca Crook:** data curation. **David Honeywell:** data curation, investigation, formal analysis. **Charlotte Lennox:** funding acquisition. **Dawn Edge:** supervision. **Patrcia Gooding:** supervision, writing – review and editing. **Gillian Haddock:** supervision, writing – review and editing. **Helen Brooks:** supervision. **Caroline Hendricks:** investigation, project administration. **Daniel Pratt:** conceptualisation.

This work was conducted at the University of Manchester and funded by the UK National Institute of Health Research (NIHR), Programme Grants for Applied Research (PGfAR) under Grant Reference Number: RP‐PG‐0218‐20006 and supported by the NIHR Manchester Biomedical Research Centre (NIHR203308). The views expressed are those of the authors and not necessarily those of the NHS, the NIHR or the Department of Health and Social Care.

## Conflicts of Interest

The authors declare no conflicts of interest.

## Data Availability

The data that support the findings of this study are available on request from the corresponding author. The data are not publicly available due to privacy or ethical restrictions. The data that support the findings of this study are available from the corresponding author upon reasonable request.
